# Supplementary material for: The genome sequence of the yellow-legged clearwing, Synanthedon vespiformis (Linnaeus, 1761)
Source: Wellcome Open Res. 2022 Sep 14;7:233. [Version 1] doi: 10.12688/wellcomeopenres.18109.1 (PMC9989545; doi:10.12688/wellcomeopenres.18109.1)
Supplement: Peer review history

# Peer review: Reviewer response for version 1

**DOI:** 10.21956/wellcomeopenres.20080.r55144

**Published:** 2023-03-06

**Authors:** Shuai Zhan

**Affiliations:** 1 University of Chinese Academy of Sciences,, Beijing, China

The authors present a chromosomal-level genome assembly for the yellow-legged clearwing *Synanthedon vespiformis*.  Presented features support that the genome reference is relatively complete in terms of gene content.  The manuscript was written clearly. I have minor concerns for the authors to improve the manuscript.

1. The method for genome assembly was not fully described. Detailed commands and parameters should be provided. More importantly, how the genome assembly was manually corrected should be described rather than linking to a previous manuscript. 
2. It's unclear to me how Chr.Z was assigned. Based on the sequencing ratio between male and female, or the cross-species synteny? Related methods and evidence should be provided.
3. (Optional) I note that the reported genome reference has not been annotated. A fully-annotated reference genome would be much more helpful for the community.

Are sufficient details of methods and materials provided to allow replication by others?

Partly

Is the rationale for creating the dataset(s) clearly described?

Yes

Are the datasets clearly presented in a useable and accessible format?

Yes

Are the protocols appropriate and is the work technically sound?

Yes

Reviewer Expertise:

genomics

I confirm that I have read this submission and believe that I have an appropriate level of expertise to confirm that it is of an acceptable scientific standard.

# Peer review: Reviewer response for version 1

**DOI:** 10.21956/wellcomeopenres.20080.r54250

**Published:** 2023-02-23

**Authors:** Jean-Michel Drezen

**Affiliations:** 1 CNRS - Université de Tours,, Tours, France

The genome report of the Lepidoptera *synanthedon vespiformis * begins with an introduction describing the natural history of the species, followed by technical aspects of genome sequencing performed to obtain high-quality genome chromosome scale assembly as in other data notes from the Darwinian tree of life project. The introduction is well written and will be of interest for a broad audience, whereas the detailed protocol and figures will be understood by specialists in genomics. The genome obtained using a combination of the best available approaches is of high quality and at chromosomal scale. The values assessing quality are all very good. This genome will provide a strong reference for future studies provided that some effort is put on the study of the genetic of mimicry that makes this and related lepidopteran species look like wasps which is quite fascinating. It will also be of interest to check for horizontal transfers of genes from their parasites, that have been shown to impact many lepidopteran genomes ^1^^,^^2^.

 By searching on the internet to find information potentially missing I did not find much to add: the scientific literature is scarce on this species. The only criticism I can provide is on the rather obscure sentence on whether the species distribution may have decreased in the UK since 1970 since as stated the sampling method now using pheromones is completely different from that used for old records. The reference cited for this sentence is not "open access" which prevent a better understanding of this point. Concerning the pest status one can also mention that it appears to be a pest also for stone fruits in Israël ^3^, which is probably one of the reasons why pheromone lures have been developed. On the other hand, the adult has probably a beneficial role as pollinator, as most Lepidoptera, as suggested by many pictures showing them foraging on flowers, but I did not find any reference on this potential role. There might be also sexual dimorphism interesting to mention but again I did not find a reference on that topic.

Are sufficient details of methods and materials provided to allow replication by others?

Yes

Is the rationale for creating the dataset(s) clearly described?

Yes

Are the datasets clearly presented in a useable and accessible format?

Yes

Are the protocols appropriate and is the work technically sound?

Yes

Reviewer Expertise:

wasp genomics, insect viruses, horizontal transfer

I confirm that I have read this submission and believe that I have an appropriate level of expertise to confirm that it is of an acceptable scientific standard.

## References

[1] : Recurrent Domestication by Lepidoptera of Genes from Their Parasites Mediated by Bracoviruses. *PLoS Genet* .2015;11(9) : 10.1371/journal.pgen.1005470 e1005470 10.1371/journal.pgen.1005470 26379286PMC4574769

[2] : Somatic chromosomal integration of polydnavirus during parasitism triggered their germline infiltration in multiple lepidopteran families. *bioRxiv* .2022; 10.1101/2022.09.22.509082 10.1101/2022.09.22.509082

[3] : Identification and field bioassays of the sex pheromone of the yellow-legged clearwing Synanthedon vespiformis (Lepidoptera: Sesiidae). *Chemoecology* .2011;21(4) : 10.1007/s00049-011-0081-7 227-233 10.1007/s00049-011-0081-7
